# Searching for the Best Transthyretin Aggregation Protocol to Study Amyloid Fibril Disruption

**DOI:** 10.3390/ijms23010391

**Published:** 2021-12-30

**Authors:** Elisabete Ferreira, Zaida L. Almeida, Pedro F. Cruz, Marta Silva e Sousa, Paula Veríssimo, Rui M. M. Brito

**Affiliations:** 1 Chemistry Department and Coimbra Chemistry Centre-Institute of Molecular Sciences (CQC-IMS), University of Coimbra, 3004-535 Coimbra, Portugal; eppferreira@gmail.com (E.F.); zalmeida@qui.uc.pt (Z.L.A.); pjfc7@ci.uc.pt (P.F.C.); sss.marta@gmail.com (M.S.e.S.); 2 Centre for Neuroscience and Cell Biology, University of Coimbra, 3004-517 Coimbra, Portugal; pverissimo@bioq.uc.pt

**Keywords:** transthyretin, protein aggregation, amyloid fibrils, amyloidosis, amyloid disruptors, fibril disaggregation, screening protocol

## Abstract

Several degenerative amyloid diseases, with no fully effective treatment, affect millions of people worldwide. These pathologies—amyloidoses—are known to be associated with the formation of ordered protein aggregates and highly stable and insoluble amyloid fibrils, which are deposited in multiple tissues and organs. The disruption of preformed amyloid aggregates and fibrils is one possible therapeutic strategy against amyloidosis; however, only a few compounds have been identified as possible fibril disruptors in vivo to date. To properly identify chemical compounds as potential fibril disruptors, a reliable, fast, and economic screening protocol must be developed. For this purpose, three amyloid fibril formation protocols using transthyretin (TTR), a plasma protein involved in several amyloidoses, were studied using thioflavin-T fluorescence assays, circular dichroism (CD), turbidity, dynamic light scattering (DLS), and transmission electron microscopy (TEM), in order to characterize and select the most appropriate fibril formation protocol. Saturation transfer difference nuclear magnetic resonance spectroscopy (STD NMR) was successfully used to study the interaction of doxycycline, a known amyloid fibril disruptor, with preformed wild-type TTR (TTRwt) aggregates and fibrils. DLS and TEM were also used to characterize the effect of doxycycline on TTRwt amyloid species disaggregation. A comparison of the TTR amyloid morphology formed in different experimental conditions is also presented.

## 1. Introduction

Currently, considerable attention is given to a group of protein misfolding diseases known as amyloidosis [1,2,3,4]. There are approximately 50 of these pathologies, involving different precursor proteins and associated with the formation of extracellular amyloid fibrils or intracellular inclusions with amyloid-like characteristics [3,5]. Consequently, amyloidoses can affect different organs, namely the heart, liver, kidneys, nervous system, spleen, skin, and gastrointestinal tract, among others. Severe amyloidosis can lead to life-threatening organ failure and death. No effective disease modifying therapies are available for most amyloid diseases, including Alzheimer’s disease (AD) and Parkinson’s disease (PD). Amyloid-β peptide, prion protein, α-synuclein, and transthyretin (TTR) are examples of the more than 40 different human peptides and proteins identified in amyloid deposits that are at the origin of the known amyloidoses [3].

Interestingly, amyloid fibrils present common morphologies and structural properties, despite being originated by quite different precursor proteins. Amyloid fibrils are long unbranched structures with a high β-sheet content structural core and thermodynamically very stable, which may be detected through binding to dyes, such as Congo red, or the fluorescent probe thioflavin-T (ThT) [1,6].

Amyloids formed by the protein transthyretin (TTR) are at the origin of several amyloidoses (ATTR) that can be divided in two main types: sporadic, age-related amyloidosis, and hereditary or familial amyloidosis. Wild-type TTR amyloidosis (ATTRwt), formerly known as senile systemic amyloidosis (SSA), is an idiopathic disease characterized by the deposition of wild-type (wt) TTR amyloid mainly in the heart. ATTRwt is strongly correlated with ageing, affecting an estimated 25% of the world population over the age of 80 [7], and may give rise to heavy deposition and infiltration of amyloid in the cardiac tissue, resulting in congestive heart failure [8]. Mutant TTR amyloidosis (ATTRm) comprises a group of familial/hereditary and rare multisystem diseases with a wide spectrum of clinical manifestations, such as familial amyloid polyneuropathy (hATTR-PN or FAP) [9], familial amyloid cardiomyopathy (hATTR-CM or FAC) [10], and familial leptomeningeal amyloidosis [11], which may occur in isolation or in combination with familial ocular amyloidosis [12] and additional clinical manifestations. These ATTRm amyloidoses are caused by more than 130 different known TTR variants [13,14].

Human TTR is a 55 kDa homotetrameric plasma protein with a high proportion of β-sheet structure that is mainly biosynthesized in the liver [15], but also synthesized in the choroid plexus of the brain [16] and in the retinal pigmentous epithelium of the eye [17]. The main known functions of TTR are the transport of thyroid hormones and retinol in association with retinol-binding protein. Additionally, TTR is also known to have a neuroprotective role [18].

Over the years, the molecular mechanisms of amyloid formation have been the subject of considerable scientific interest [19,20]. In vitro fibril formation has been a powerful tool in the study of the mechanism that leads to amyloid formation, helping to explain the events occurring in vivo. Regarding the TTR aggregation process, TTR instability caused by mutations or by other factors, leads to the dissociation of the native tetramer into monomers that undergo partial unfolding [21,22,23]. These non-native monomers can associate into soluble oligomers that self-assemble, leading to the final amyloid fibril [20,24]. However, the clinical and genetic heterogeneity of ATTR, together with the different structural and functional characteristics of the TTR variants, suggests that TTR fibril formation may involve diverse molecular pathways [25]. Amyloid deposits may be constituted not only by full-length TTR fibrils, but also by C-terminal TTR fragments and amorphous aggregates [26,27,28].

Although liver transplantation and therapies [29,30] such as TTR stabilization [27] or suppression of TTR expression [31,32,33,34,35,36] are currently in use, there is no fully effective treatment for all the clinical manifestations of ATTR amyloidosis yet. Considering the current knowledge of TTR and TTR amyloid formation, various therapeutic strategies targeting different steps of the aggregation pathway may be devised [28,29,30]. The disruption of amyloid aggregates and fibrils is one of these strategies, and has gained importance in both experimental assays and clinical studies [37,38,39,40,41,42,43,44,45,46] in the last two decades. However, to date, only a few compounds have been identified as possible amyloid fibril disruptors for ATTR. Among these are some polyphenols, such as EGCG (epigallocatechin-3-gallate) [37,38,45], IDOX (4′-iodo-4′-deoxydoxorubicin) [39,40], and doxycycline [42,43,46].

In order to discover new amyloid fibril disruptors against ATTR, we aimed to develop an appropriate in vitro screening protocol for TTRwt disaggregation. For this purpose, the first step was the characterization and selection of an appropriate aggregation protocol and identification of the respective amyloid aggregate/fibril model. Ideally, the in vitro formed amyloid species would have similar structure and morphology to in vivo formed species, and the aggregation protocol experimental conditions would be as close as possible to the in vivo process of TTR amyloid formation, maintaining physiological conditions, such as average protein concentration, pH, ionic strength, and temperature. However, the native tetrameric form of TTRwt has high conformational stability [47,48,49], and the amyloid formation process in these conditions is lengthy and thus is unsuitable to be used in screening protocols. After a careful analysis of the available literature [21,50,51], three aggregate and fibril formation protocols were selected for comparison: acidification at pH 2.0 and pH 4.4, and heating at pH 7.4. Furthermore, the effect of changing the pH from 2.0 or 4.4 to physiological conditions (pH 7.4), after aggregation, was also studied. Thioflavin-T (ThT) fluorescence assays, circular dichroism (CD), turbidity, dynamic light scattering (DLS), and transmission electron microscopy (TEM) were used to characterise the timing and products formed with these protocols and the effect of pH modification. The choice of appropriate experimental conditions and methods to provide relevant information on the effect of particular compounds on fibril interaction and disaggregation is of paramount importance in a screening protocol.

Saturation transfer difference nuclear magnetic resonance spectroscopy (STD NMR) was used to study the interaction of TTR fibrillar structures and a known TTR fibril disruptor—doxycycline—in more detail. DLS, TEM, and turbidity experiments were used to characterize the effect of this compound on TTR amyloid fibril disruption. Additionally, doxycycline was chosen to validate the experimental protocols due to its well demonstrated anti-amyloidogenic activity in vitro [46] and in vivo. It has been shown that doxycycline acts as L55P TTR amyloid fibril disruptor in vitro [40] and disaggregates amyloid deposits in V30M TTR transgenic mice with a concomitant decrease of various amyloidosis tissue markers [41,42]. This compound was even used in clinical trials, sometimes in combination with TUDCA (tauroursodeoxycholic acid) [43] and other times in combination with UDCA (ursodeoxycholic acid) [52].

## 2. Results

### 2.1. Characterization of TTRwt Fibril Formation Protocols

In order to summarize the three aggregation protocols of TTRwt used in this study, Table 1 shows the main requirements for these protocols in terms of experimental conditions, and Table 2 summarizes the main characteristics of the aggregates and fibrils formed during aggregation and the result of pH changes on these amyloid structures.

**Figure 1:**
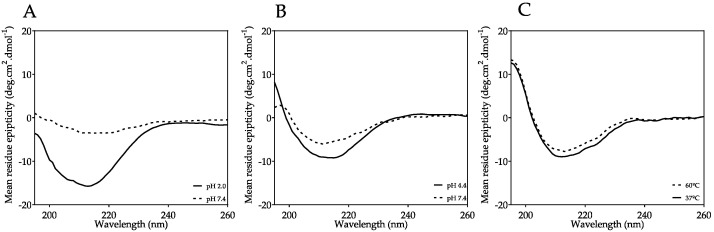
Far-UV CD spectra of TTRwt fibrils from different fibril formation protocols. (**A**) TTRwt fibrils assembled at pH 2.0 and after pH adjustment to 7.4, (**B**) at pH 4.4 and after pH adjustment to 7.4, and (**C**) at pH 7.4 at 60 °C and after temperature change to 37 °C. All TTRwt samples were analysed at 3.6 μM and all CD spectra were acquired at the end of each fibril formation protocol.

**Figure 2:**
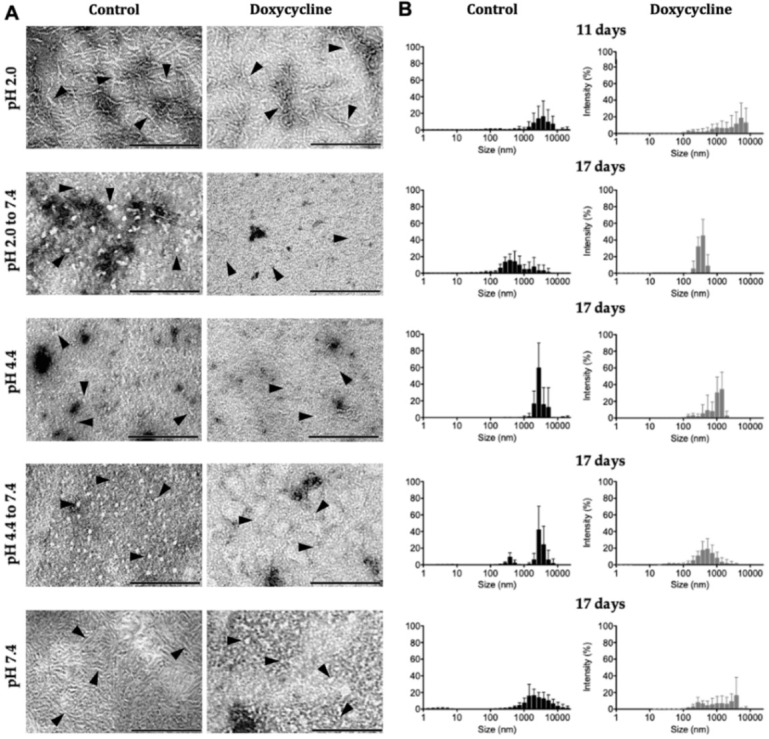
Analysis of the effect of doxycycline on preformed TTRwt aggregates and fibrils. TTRwt amyloid species (3.6 μM) assembled at pH 2.0 (pH 2.0), pH 2.0 followed by pH adjustment to 7.4 (pH 2.0 to 7.4), pH 4.4 (pH 4.4), pH 4.4 followed by pH adjustment to 7.4 (pH 4.4 to 7.4), and induced by heating at pH 7.4 (pH 7.4) were incubated both in the absence (control) and presence of 50× molar excess of doxycycline (180 μM) for the indicated period of time and analysed by (**A**) TEM and (**B**) DLS. The scale bars represent 200 nm, and the arrows indicate some of the amyloid structures observed.

According to Table 1 and Table 2, the fibril formation protocol performed at pH 2.0 with 0.1 M NaCl was more time-consuming and required higher concentrations of protein. Amyloid fibrils observed by TEM appeared as long unbranched filamentous structures 5 to 9 nm in diameter with variable length, indicating a mature growth of these fibrillar structures [1] which were ThT positive. The far-UV CD spectrum also showed a minimum around 213 nm, and a CD spectrum typical of TTRwt amyloid fibrils [20] with high β-sheet content (Table 3). In an attempt to mimic the in vivo conditions of TTRwt amyloid fibrils, pH adjustment to pH 7.4 of preformed fibrils at pH 2.0 was carried out, and the effect on fibril size, structure, and morphology was analysed by DLS, CD, and TEM, respectively. DLS results showed that after 11 days of fibril incubation at pH 7.4, the sample had larger populations of smaller particles (between 100 and 1000 nm) compared to fibril incubation at pH 2.0 (Figure S1). The changes in secondary structure of preformed TTRwt fibrils were analysed by far-UV CD and the minimum at 213 nm was not as pronounced, indicating a clear loss in β-sheet and a gain of unordered secondary structures (Figure 1A and Table 3). TEM observations indicated that, at pH 7.4, fibrils lost their extended fibrillar structure and became more spherical and amorphous with diameters between 4 and 13 nm when compared to fibrils at pH 2.0 (Figure 2A, pH 2.0 to 7.4), corroborating DLS and CD results; however, they still remained ThT positive.

Regarding the aggregation at pH 4.4, this protocol was the least time-consuming, had low protein requirements, and was suitable for pH adjustment to 7.4 (Table 1 and Table 2). The TEM images of the amyloid structures formed showed shorter fibrils that were 5 to 6 nm in diameter and at least 25 nm in length, as well as some amorphous aggregates and several oligomeric species that were 4 to 6 nm in diameter (Figure 2A, pH 4.4). The CD spectrum also indicated a less pronounced β-sheet profile, corroborating the TEM results (Figure 1B and Table 3). In addition, the DLS and CD experiments showed that pH adjustment to 7.4 slightly modified the particle size (Figure S2 and Figure 2B, pH 4.4 to 7.4) and secondary structure (Figure 1B). The TEM images revealed more spheroid and amorphous morphologies and less fibrillar structures (4 to 5 nm in diameter, and up to 25 nm in length), which is in total agreement with a small loss of β-sheet structure and an increase in unordered structure (Table 3) when compared with the fibrils formed at 4.4, and even at pH 2.0 (Figure 2A, pH 4.4 to 7.4); however, they remained ThT positive (Table 2).

Regarding the heat-induced fibril formation at pH 7.4, this protocol had a duration of 6 days and required low concentrations of protein (Table 1). The resulting species were ThT positive (Table 2), and TEM images showed unbranched fibrillar structures that were shorter and narrower than the fibrils formed at pH 2.0, with a diameter of 4 to 6 nm and a length of 10 to 50 nm (Figure 2A, pH 7.4). The CD spectrum also displayed the typical β-sheet profile (Figure 1C and Table 3). To better characterize this protocol, turbidity was also used to follow the aggregation process. An increase in turbidity was observed in the 6 days of heating (Figure S3), which implies aggregation. The transition from 60 °C to 37 °C did not induce a decrease in turbidity, indicating that the decrease of temperature neither causes fibril breakdown (Figure S3, 8th day) nor an alteration in secondary structure (Figure 1C and Table 3).

### 2.2. Interaction of Doxycycline with Preformed TTRwt Fibrils

**Figure 3:**
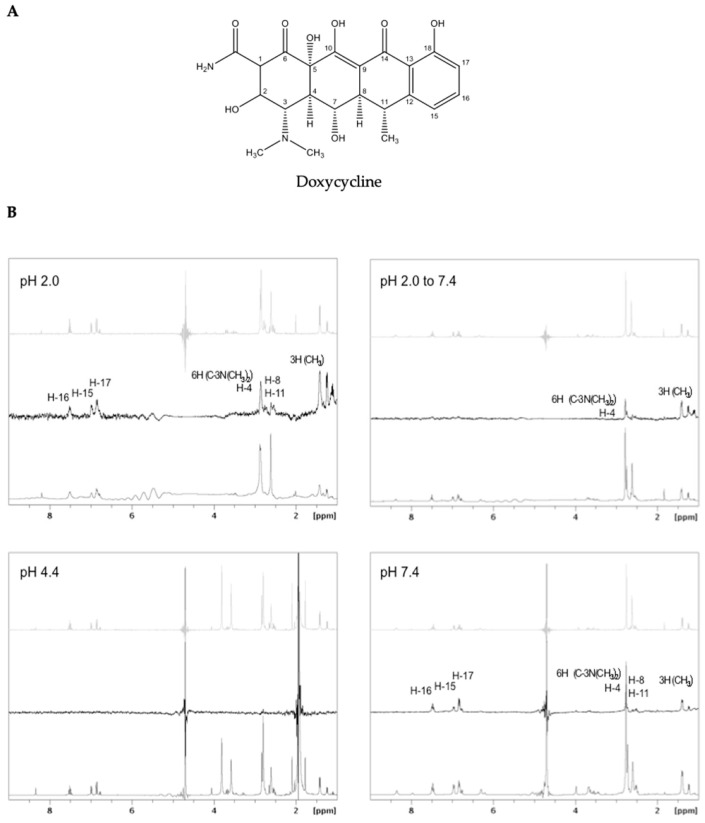
(**A**) Molecular structure of doxycycline and (**B**) ^1^H STD NMR of doxycycline (2 mM) in the presence of preformed TTRwt fibrils (20 μM) assembled at pH 2.0 (pH 2.0), pH 2.0 that underwent pH adjustment to 7.4 (pH 2.0 to 7.4), pH 4.4 (pH 4.4), and assembled by heating at pH 7.4 (pH 7.4). The reference ^1^H NMR spectrum of doxycycline at the corresponding pH is represented in light grey, the ^1^H STD NMR spectrum is represented in black, and the off-resonance spectrum is represented in grey.

The interaction between doxycycline (Figure 3A) and preformed TTRwt aggregates and/or fibrils, prepared according to the previously described aggregation protocols, was studied using ^1^H STD NMR. The ^1^H NMR spectrum of doxycycline (Figure 3B, light grey), ^1^H STD NMR spectrum of doxycycline in the presence of TTRwt fibrils (Figure 3B, black), and the off-resonance spectrum (Figure 3B, grey) for each experimental condition is represented in Figure 3B. The signals present in the STD spectrum, identified by their chemical shifts, represent the doxycycline protons that are in close contact with TTR amyloid structures.

At pH 2.0, the doxycycline signals from the aromatic protons (i.e., H-15, H-16, and H-17), three methyl groups, and protons H-8, H-11, and H-4 were observed to interact with TTRwt amyloid fibrils (Figure 3B, pH 2.0). However, additional signals between 2.5 and 3 ppm could not be unambiguously assigned due to the high density of resonances in this region. Moreover, the broadening of the ligand signals in the presence of fibrils is due to alteration of the relaxation time, and therefore is evidence of binding [53]. When the pH of the fibrils formed at pH 2.0 was adjusted to 7.4, the signals from the aromatic protons disappeared, except the proton H-4 and protons from the three methyl groups, as can be seen in the corresponding STD spectrum (Figure 3B, pH 2.0 to 7.4).

Doxycycline proton signals were absent in the ^1^H STD NMR spectrum of doxycycline in the presence of TTRwt amyloid structures at pH 4.4 (Figure 3B, pH 4.4). Contrary to what occurred with the other aggregated samples prepared at pH 2.0 with 0.1 M NaCl and pH 7.4 at 60 °C, the sample with TTRwt aggregates and fibrils assembled at pH 4.4 was extremely turbid, and the amyloid content seemed to quickly precipitate in the NMR tube during the assay.

At pH 7.4, the doxycycline protons involved in the interaction with the heat-induced fibrils were similar to those at pH 2.0 (Figure 3B, pH 7.4).

### 2.3. Effect of Doxycycline on TTRwt Fibril Disaggregation

The effect of doxycycline on fibril disruption was assessed by DLS, TEM, and in some of the aggregation protocols by turbidity. Preformed TTRwt aggregates and fibrils were incubated both in the absence and presence of a 50× molar excess of doxycycline relative to tetrameric TTRwt. DLS measurements were carried out every day until the apparent size stabilization of the species in solution was achieved. TEM images were acquired prior to doxycycline addition and after treatment with doxycycline when the sizes of the particle populations (assessed by DLS) had stabilized. Doxycycline, at 50× the molar concentration of TTRwt, did not have an immediate persistent effect on the aggregates/fibrils prepared previously with the different protocols, taking from 11 to 17 days to reach a maximum observable disruptive effect.

At pH 2.0, after 11 days of incubation with doxycycline, fibrils showed a maximum length of 130 nm and were 4 to 7 nm in diameter, which was a clear decrease of the fibril length and diameter observed by TEM, indicating fibril disruption (Figure 2A, pH 2.0). Moreover, DLS results also supported this observation, which was visible as an increase in particle populations between 100 and 1000 nm (Figure 2B, pH 2.0). Turbidity assays were also carried out to follow TTRwt amyloid fibril aggregation and disaggregation in the presence of doxycycline. However, no signal in turbidity intensity was observed, both in treated and untreated samples with doxycycline (data not shown). This result may be explained by the formation of a clear and completely transparent solution, thus implicating no light scattering signal detection in the pH 2.0 samples, especially when compared to turbid solutions as is the case of pH 4.4 samples.

When the fibrils assembled at pH 2.0 underwent pH adjustment to 7.4 and were then treated with doxycycline, the disruptive effect was visible after 17 days both by TEM, with a decrease in the size of the non-fibrillar structures with diameters of 4 nm (Figure 2A, pH 2.0 to 7.4), and DLS, with the disappearance of particle populations equal to or larger than 1000 nm (Figure 2B, pH 2.0 to 7.4).

**Figure 4:**
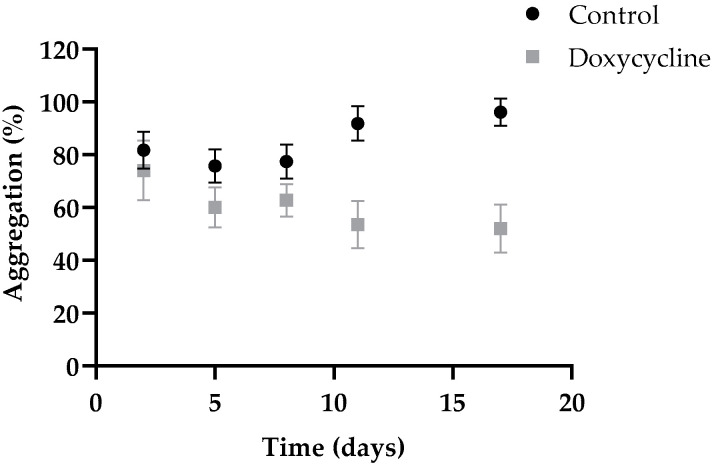
Effect of doxycycline on preformed TTRwt fibrils at pH 4.4 monitored by turbidity. Samples of preformed TTRwt fibrillar species (3.6 μM) were incubated both in the absence (control) and presence of 50× molar excess of doxycycline (180 μM) for the indicated time, at room temperature.

The disruptive effect of doxycycline was also observed in the pre-assembled aggregates/fibrils at pH 4.4 after 17 days of incubation. A decrease in the size of fibrillar structures following treatment with doxycycline was observed by TEM (maximum of 15 nm in length and diameter between 3 and 4 nm) (Figure 2A, pH 4.4). DLS results showed that the control sample had sizes ranging between 1000 and 10,000 nm, whereas in the doxycycline-treated sample, a decrease in the larger size particle populations and an increase in the smaller size populations between 100 and 1000 nm was noticeable (Figure 2B, pH 4.4). Additionally, a turbidity assay was also performed to further characterize the disruptive effect of doxycycline over time in preformed amyloid material (Figure 4). A more pronounced decrease in the turbidity of doxycycline-treated samples was observed at day 11, demonstrating that the aggregated species in solution were smaller or/and in smaller amount. This effect increased in the following days, reaching a maximum at day 17 with an aggregation estimate of approximately 50% of the control.

When the pre-assembled aggregates and fibrils at pH 4.4 underwent pH adjustment to 7.4 and were treated with doxycycline, the disruptive effect was also observed. After 17 days of incubation with doxycycline, the aggregates were 4 to 5 nm in diameter, as observed by TEM images. Moreover, although the fibrillar structures maintained their approximate diameter (4 nm) and length (maximum of 25 nm), they had a lower frequency of occurrence when compared to the untreated samples (Figure 2A, pH 4.4 to 7.4). The DLS results showed that, at this time, the particle populations of doxycycline-treated samples were comprised of particles with sizes between 100 and 1000 nm, whereas in the control samples the majority of the particle populations were higher than 1000 nm, which supports a fibril disaggregation mechanism (Figure 2B, pH 4.4 to 7.4).

The disruptive effect of doxycycline was also observed in the preformed heat-induced TTRwt fibrils at pH 7.4. The TEM images acquired after 17 days of treatment showed fibrillar structures exhibiting diameters between 3 to 4 nm and lengths between 10 to 25 nm, and a change in morphology to more amorphous structures in some cases (Figure 2A, pH 7.4). At this incubation time, the DLS results showed the doxycycline-treated samples were comprised of particles on average smaller, with sizes between 100 and well below 10,000 nm, whereas in control samples with no doxycycline the major particle populations were above 1000 nm (Figure 2B, pH 7.4). Thus, doxycycline also induces fibril disruption of heat-induced TTRwt fibrils. Turbidity assays were also performed in order to follow TTRwt amyloid fibril disaggregation in the presence of doxycycline. However, the results showed a slight increase in the turbidity intensity, when compared to control samples (data not shown), which might be explained by the large amount of smaller species formed during the disaggregation induced by doxycycline, in particular amorphous species, as mentioned previously, leading to higher light scattering intensity when compared to the short and unbranched fibrillar structures observed in the control sample (Figure 2A, pH 7.4).

## 3. Discussion

With the goal of finding an effective and relatively fast screening protocol to search for TTR amyloid fibril disruptors, we have reviewed, characterized and selected the most appropriate TTR aggregation protocol among those previously described. After a careful analysis of the available literature, three TTRwt aggregation protocols were selected for comparison, performed at different pH values: pH 7.4, pH 4.4, and pH 2.0 (Table 1 and Table 2). The protocol involving heat induction at pH 7.4 was selected since it is performed at the pH of physiological conditions [51]. Acidification is the most widely used strategy to induce TTR aggregation. At pH 4.4 the TTR tetrameric structure dissociates into monomeric species that undergo partial unfolding and, consequently, associate to form amyloid structures [21]. Acidification at pH 2.0 promotes tetramer dissociation followed by denaturation, and the addition of 0.1 M NaCl induces aggregation, since chloride ions shield the positive charges of the protein, allowing for conformational changes and the assembly of monomers into oligomers and amyloid fibrils [54]. Thus, aggregation processes are extremely dependent not only on pH changes, but also on protein concentration, ionic strength, temperature, and agitation.

The aggregation protocol of TTRwt at pH 2.0 with 0.1 M NaCl has been previously well characterized and its kinetics studied by our research group [20]. This is the most time-consuming of the three protocols, and it requires a relatively high concentration of tetrameric TTRwt to be effective in an acceptable time period. Aggregation at this pH, with a physiological plasma protein concentration (3.6 μM), was also performed and the process was followed by DLS for approximately 30 days. However, no significant changes in particle populations were observed during the experiment (data not shown), which indicates that DLS could not detect fibril formation in this period of time at 3.6 μM TTR. The fibrils formed at pH 2.0 are unbranched, long, well-formed and mature (Figure 2A, pH 2.0), and bind ThT (Table 2), a good indication of presence of amyloid structures. Adjustment of pH to 7.4 of TTRwt fibrils preformed at pH 2.0 was an attempt to mimic the in vivo conditions of TTRwt fibrils and to be able to study the action and efficacy of potential disruptors in close to physiological conditions. TTRwt amyloid deposits are mostly extracellular [55] where pH is generally close to neutral. The effect of pH modification on the size, structure, and morphology of preformed TTRwt amyloid was studied by DLS, CD, and TEM experiments, respectively. The DLS results showed that incubation in phosphate buffered saline (PBS), at pH 7.4, of TTRwt amyloid fibrils preformed at pH 2.0 led to a decrease in the average size of the particles, with populations of particles smaller than 1000 nm being more common (Figure S1B). The CD results revealed a loss of β-sheet secondary structure (Figure 1A and Table 3), and the TEM images corroborated the previous observations showing that fibrils lose their morphology, appearing as smaller fibrils and amorphous aggregates (Figure 2A, pH 2.0 to pH 7.4); however, they still remained ThT positive (Table 2).

The fibril formation protocol at pH 4.4 is widely used for in vitro TTRwt amyloid fibrillogenesis inhibitor screening, as it is easily implemented in medium or high throughput screening formats in tubes or microwell plates, usually followed by turbidity measurements [56,57,58]. Thus, this protocol seems to be a very good candidate for use in TTRwt disaggregation assays. This method is less time-consuming than the previous method at pH 2.0, does not require high concentrations of native TTRwt, and amyloid species can be formed at physiological concentrations (3.6 μM) after only 3 days (Figure S4). The formation of amyloid aggregates and fibrils using this protocol can be monitored by both DLS and turbidity. In an attempt to follow aggregation, CD was also performed every day for 3 days; however, the spectra remained unaltered and similar to Figure 1B. At pH 4.4, the species formed are composed by small fibrils and aggregates, while samples remained ThT positive (Table 2). pH adjustment to 7.4 was also performed for these amyloid structures, with CD (Figure 1B), DLS (Figure 2B, pH 4.4 to pH 7.4), and TEM (Figure 2A, pH 4.4 to pH 7.4) assays demonstrating that the pH change slightly altered the structure, size, and morphology of these aggregates; however, they remained ThT positive (Table 2).

The third aggregation protocol is the least studied process of the three. Heat-induced fibril formation at pH 7.4 seems to be a very good candidate for use in TTRwt fibril disruptor screening assays because avoids the influence of low pH on the mechanism of action of the disruptor. Our experiments showed that this protocol is not very time-consuming, it occurs at physiological concentrations, and the unbranched fibrils are formed and maintained at physiological pH for at least 6 days (Figure S5). The fibril formation process was followed by DLS (Figure 2B, pH 7.4), turbidity (Figure 4), and CD (Figure 1C) assays. The increase in turbidity observed over the 6 days of incubation at 60 °C (Figure S3), the increase in particle populations with sizes between 1000 and 10,000 nm, and the decrease in particle populations of smaller sizes observed by DLS demonstrate aggregation (Figure S5). This process was not reverted by temperature adjustment to 37 °C, as evidenced by absence of changes in turbidity and particle populations. The far-UV CD spectra and secondary structure predictions also showed presence of high β-sheet content during the 6 days of incubation at 60 °C, or after a temperature change to 37 °C, indicating no major changes in the characteristic β-sheet secondary structure of TTRwt amyloid fibrils (Figure 1C and Table 3). The temperature-induced fibrils were ThT positive (Table 2), which is a clear indication of amyloid structures.

In order to follow amyloid TTRwt disaggregation in this study, a well-known amyloid fibril disruptor was selected to serve as a positive control. There are only a few amyloid disruptor compounds that have already been tested with TTR, and doxycycline is one of them. In addition, doxycycline has also been tested with other amyloid protein precursors, such as amyloid-β peptide, polyglutamine, β-2-microglobulin, islet amyloid polypeptide, and immunoglobulin light chain, among others. Thus, it seems that doxycycline is a small molecule with the ability to bind not only several amyloid systems, but also different amyloid structures, as also observed with amyloid dyes thioflavin-T and Congo red [1].

^1^H STD NMR experiments were used to probe the interaction between preformed TTRwt aggregates/fibrils and doxycycline, since it is a well-known methodology for studying intermolecular interactions. This technique allows the identification of the regions of the small molecule (epitopes) mostly involved in the interaction with the amyloid structures, which may be important for the discovery, development or selection of other potential small molecule disruptors. Doxycycline seems to be relatively stable at pH 2.0, 4.4, and 7.4. The ^1^H NMR spectra of doxycycline at these pH values (Figure S6) do not show significant changes in the doxycycline proton signals, which is indicative of absence of chemical modification or degradation. Different concentrations of preformed TTRwt fibrils were tested in ^1^H STD NMR preliminary experiments at pH 2.0, and 20 μM was the minimum protein concentration detectable. This protein concentration was used with 100× molar excess of doxycycline to reach the STD regime and obtain the most information from all ^1^H STD NMR experiments.

When analysing the ^1^H STD NMR spectra, it is noticeable that doxycycline interacts differently with amyloid species with distinct morphologies obtained with different TTRwt aggregation protocols (Table 2), and with aggregates or fibrils that undergo a pH change. The doxycycline hydrogen atoms involved in the interaction with preformed TTRwt fibrils assembled at pH 2.0 are aromatic hydrogens and hydrogens from the three methyl groups (Figure 3B, pH 2.0). When these preformed fibrils at pH 2.0 underwent pH adjustment to 7.4, signals from the aromatic protons were not present on the ^1^H STD NMR spectrum, indicating that they were not involved in the interaction. As previously described, the pH transition from pH 2.0 to pH 7.4 induces alterations in the morphology and structure of the fibrils, which may cause changes in the protonation states of acidic and basic residues and, consequently, modify the interactions between fibrils and doxycycline. In the ^1^H STD NMR spectrum of doxycycline in the presence of preformed TTRwt aggregates/fibrils assembled at pH 4.4, doxycycline proton signals were absent (Figure 3B, pH 4.4). ^1^H STD NMR can only be used to probe low affinity interactions (dissociation constant, K_D_, ranging from 10^−8^ M to 10^−3^ M) [59]. Therefore, this result might be attributed to absence of binding, or a very weak or strong binding of doxycycline to the fibrils, outside the detection limits of STD-NMR. In subsequent assays using DLS and TEM, it was observed that doxycycline disrupted TTRwt fibrils assembled at pH 4.4 (Figure 2A and Figure 3B, pH 4.4); thus, the no binding hypothesis can be discarded. Another observation to consider is that the TTRwt aggregated samples at pH 4.4 for NMR analysis were extremely turbid, and aggregated species precipitated in the NMR tube, which would clearly affect and explain the STD NMR negative result. The doxycycline protons involved in the binding of preformed heat-induced fibrils at pH 7.4 (Figure 3, pH 7.4) are the same as those involved in the binding with preformed TTRwt fibrils assembled at pH 2.0 (Figure 3, pH 2.0). The fibrils assembled employing these two methods were morphologically and structurally very similar, as demonstrated by TEM (Figure 2A, pH 2.0 and Figure 2A, pH 7.4) and CD (Figure 1A,C and Table 3), respectively, in agreement with the ^1^H STD NMR results. 

Herein, the disruptive effect of doxycycline on preformed TTRwt aggregates and fibrils was evaluated for the first time in vitro. In previous studies, the effect of doxycycline as an amyloid fibril disruptor has been tested on L55P and V30M TTR fibrils [42,46,60]. Doxycycline, at a molar ratio of 50× relative to TTRwt, does not have an immediate effect on TTRwt fibrils, taking 11 to 17 days to reach a maximum disruptive effect (Figure 2), indicating very slow kinetics of disaggregation. Although the compound’s effect on preformed amyloid fibrils formed at pH 2.0 and at pH 7.4 appears to be very similar, according to DLS measurements (similar particle populations) (Figure 2B), TEM images show some differences concerning size and morphology of species formed after disaggregation (Figure 2A). In addition, in vivo assays have demonstrated that doxycycline is more efficient in disaggregating well-formed fibrils [42]. In the present study, this effect was also visible since doxycycline had a higher disruptive effect on mature fibrils formed at pH 2.0 (11 days) than on aggregates or shorter amyloid fibrils formed at pH 4.4 or pH 7.4 (17 days), respectively. In fibrils assembled at pH 2.0 that underwent pH adjustment to 7.4, the doxycycline disruptive effect was also noticeable. In this case, the particle populations of higher sizes decreased; however, new populations of smaller particles did not seem to appear (Figure 2, pH 2.0 to 7.4). At pH 4.4, doxycycline also had a disruptive effect, which could be observed by DLS (Figure 2B, pH 4.4), TEM (Figure 2A, pH 4.4), and turbidity (Figure 4). Furthermore, when the pH was changed to 7.4, the disruptive effect was also very clear (Figure 2, pH 4.4 to 7.4). As previously discussed, the diverse effects of doxycycline on the TTRwt aggregates and fibrils prepared by different aggregation protocols may be attributed to the pH of the sample, or more likely to the structural differences between amyloid species.

## 4. Materials and Methods

### 4.1. Reagents

Doxycycline was purchased from AK Scientific (Union City, CA, USA), and hydrochloric acid was obtained from Merck (Frankfurt, Germany). Sodium azide, thioflavin-T (ThT), deuterium oxide (D_2_O), deuterated dimethyl sulfoxide (DMSO-*d_6_*), DMSO, and all other reagents used for buffer and sample preparation were purchased from Sigma-Aldrich (St. Louis, MO, USA), and were obtained with the highest purity available.

### 4.2. Samples

Recombinant wild-type TTR (TTRwt) was produced in a bacterial expression system and purified as previously described [20]. TTR tetramer concentration was determined by UV-vis spectroscopy with a UV500 Spectronic Unicam (Cambridge, UK) spectrophotometer by examining the absorption at 280 nm and using its molar absorption coefficient (ε_280_ = 7.76 × 10^4^ M^−1^ cm^−1^) [22].

Doxycycline solutions were prepared in DMSO or in DMSO-d_6_. The final concentration of DMSO in samples for turbidity, dynamic light scattering (DLS), and transmission electron microscopy (TEM) experiments was 1%, and the final concentration of DMSO-*d_6_* in saturation transfer difference nuclear magnetic resonance (STD NMR) experiments was 4%.

### 4.3. Amyloid Fibril Formation Protocols

For the preparation of TTRwt fibrils at pH 2.0, samples of acid-unfolded TTR were prepared by dialysis against 10 mM HCl, pH 2.0, for 96 h at 4 °C. TTR aggregation was induced by addition of NaCl to a final concentration of 0.1 M [20,54]. Aggregation was carried out for at least a week at room temperature (25 °C). The concentration of tetrameric TTRwt used was approximately 80 μM, and was diluted to 3.6 μM for the experimental assays at the same temperature.

Preformed TTRwt fibrils assembled at pH 2.0 with 0.1 M NaCl were diluted in phosphate buffered saline (PBS) at pH 7.4 to 3.6 μM, in order to induce a pH change from 2.0 to 7.4, and maintained at 25 °C.

To prepare TTRwt fibrils at pH 4.4 and 3.6 μM, aggregation was induced by addition of TTRwt in 10 mM sodium phosphate, 100 mM KCl, and 1 mM EDTA (pH 7.2), to an equivalent volume of 200 mM sodium acetate, 100 mM KCl, and 1 mM EDTA (pH 4.32) [21]. The resulting solution at pH 4.4 was incubated for 72 h at 37 °C. Preformed TTRwt fibrils were then maintained at 25 °C.

To induce a pH change from 4.4 to 7.4, preformed TTR fibrils assembled at pH 4.4 were diluted five times in 50 mM Tris-HCl buffer at pH 8.7 to a final TTRwt concentration of 3.6 μM and were maintained at 25 °C.

For fibril formation at pH 7.4, TTRwt at 3.6 μM in 2 mM sodium phosphate and 20 mM NaCl, pH 7.4, was heat-induced at 60 °C for 6 days [50]. Preformed TTRwt fibrils were then maintained at 37 °C.

### 4.4. Thioflavin-T Assay

A thioflavin-T (ThT) fluorescence assay was used to probe amyloid formation in all TTRwt aggregation protocols. A stock solution of ThT in 5 mM glycine–NaOH buffer, pH 9.0, was added to protein solutions at 3.6 μM to a final concentration of 10 μM. The ThT stock solution was previously filtered through 0.2 μm syringe filters, and its concentration was determined by UV-vis spectroscopy by measuring the absorption at 411 nm and using the respective ThT molar absorption coefficient (ε_411_ = 2.2 × 10^4^ M^−1^ cm^−1^) [61].

Fluorescence experiments were performed with a Varian Cary Eclipse spectrofluorometer (Varian Ltd., Surrey, UK) equipped with a temperature controller system and monitored by the Varian Cary Eclipse software version 1.1. Assays were conducted in 5 × 5 mm pathlength cuvettes with 5 nm of excitation and emission slits and continuous agitation with a magnetic stirrer. ThT fluorescence spectra were collected between 460 and 560 nm with an excitation wavelength of 450 nm at 25 °C. Baseline correction was performed by subtracting the spectrum of the buffer solution with ThT from the corresponding raw data.

### 4.5. Circular Dichroism Spectroscopy (CD)

Far-UV CD data were acquired with an Olis DSM 20 circular dichroism spectropolarimeter (OLIS, Inc., Bogart, GA, USA) continuously purged with nitrogen, equipped with a Quantum Northwest CD 150 temperature controller system (Quantum Northwest, Inc., Liberty Lake, WA, USA) and managed by the GlobalWorks software version 4.3 provided by OLIS, Inc. Depending on the amyloid fibril formation protocol, scans were collected between 195–260 nm, with increments of 1 nm at 25 °C, 37 °C, or 60 °C, and three consecutive scans with an integration time of 5 s per nm were averaged. The protein concentration used was 3.6 μM and the cuvette pathlength was 0.2 mm. A baseline correction was performed by subtracting the spectrum of the buffer solution from the raw CD data.

The secondary structure content of the CD spectra was estimated using the web server BeStSel (http://bestsel.elte.hu, accessed multiple times in 2021) [62].

### 4.6. Turbidity Assays

Absorbance measurements in turbidity assays were performed in 96-well microplates at 450, 500, 550, 600, and 650 nm, at 25 °C or 37 °C according to the experimental assay, with a microplate spectrophotometer (BioTek Instruments, Inc., Winooski, VT, USA) managed by the Gen 5 software version 2.01 provided by BioTek Instruments, Inc. The plates were linearly agitated for 30 s before the measurement.

Samples were prepared to a final volume of 100 μL in each well. All assays were performed in triplicate. The absorption values of the negative controls were determined and subtracted from the corresponding raw data. The final percentage of aggregation was calculated as an average of the wavelengths measured and represented with the corresponding standard deviation. The extent of amyloid fibril formation was assigned as 100% at the end of the TTRwt aggregation experiment.

### 4.7. Saturation Transfer Difference Nuclear Magnetic Resonance (STD NMR)

All NMR measurements were performed with a Bruker Avance III spectrometer (Bruker BioSpin Corp., Wissembourg, France) operating at a ^1^H frequency of 400.133 MHz at 25 °C or 37 °C, depending on the aggregation protocol.

NMR samples were prepared in Shigemi tubes (Shigemi Co., LTD., Tokyo, Japan) containing 20 μM of preformed TTRwt fibrils in the corresponding buffer, 90% H_2_O/10% D_2_O (*v/v*), and a 100× molar excess of doxycycline relative to TTRwt as tetramer.

To saturate the protein selectively, a total saturation time of 5 s was applied, consisting of a pulse train of 40 Gaussian bell-shaped selective pulses of 50 ms in length each, separated by a 1 ms delay. A 10 ms spin lock pulse (T1ρ) was used to remove residual protein resonances. Difference spectra were obtained using a frequency list for on-resonance and off-resonance irradiation. Water suppression was performed by excitation sculpting. All data were processed and analysed with the Bruker software TopSpin v2.1.

### 4.8. Dynamic Light Scattering (DLS)

To study the effect of doxycycline on TTRwt fibril disaggregation, preformed TTRwt aggregates/fibrils (3.6 μM) were incubated with and without doxycycline (180 μM, corresponding to a 50× molar excess relative to TTRwt), and with 0.02% sodium azide to prevent microorganism growth. The DLS measurements were carried out in 5 × 5 mm pathlength cuvettes and, according to the experimental assay, were performed at 25 °C, 37 °C, or 60 °C, with a N5 Submicron Particle Size Analyzer (Beckman Coulter, Miami, FL, USA) controlled by PCS software. Each sample was gently agitated and measured ten times with an equilibration time of 5 min and an acquisition time of 90 s. The sample volume used in these assays was 300 μL.

All the buffer solutions were previously filtered through 0.2 μm syringe filters (Whatman, UK).

### 4.9. Transmission Electron Microscopy (TEM)

The disruptive effect of doxycycline was also analysed using TEM on preformed TTRwt amyloid content (3.6 μM) with and without 50× molar excess of doxycycline (180 μM) relative to TTRwt, and 0.02% sodium azide. The sample aliquots (5 μL) were adsorbed onto carbon coated collodium films supported on 400-mesh copper grids for 1 min. The grids were negatively stained with 1% uranyl acetate and visualized using a FEI-Tecnai G2 Spirit Biotwin transmission electron microscope equipped with a SIS CCD camera MegaView III.

The analyses of TEM images were performed using the ImageJ software [63] to determine the length and diameter of the aggregates and fibrils formed during aggregation and disaggregation assays.

## 5. Conclusions

Until recently, there were few treatment options for patients with TTR amyloidosis (ATTR). Even today, no treatments are available to effectively address all the clinical manifestations associated to ATTR. The development of new therapeutic approaches is crucial to determine the optimal treatment for a given patient with ATTR, since this is a complex disease with multiple clinical manifestations, including unmet medical needs and heterogeneity of incidence and onset. Therefore, potential alternative therapies should aim at the disaggregation of already formed amyloid aggregates present in several tissues in ATTR patients. In the last two decades, this strategy of amyloid disaggregation has gained interest, and several compounds are being currently tested in vitro and in vivo with such purpose, either with TTR or other amyloid protein precursors.

Bearing in mind the screening of compounds capable of disrupting preformed TTR amyloid structures in vitro, in this study we developed and characterized a screening protocol to identify the activity of potential disruptors of aggregates and fibrils of TTRwt. As observed in the results reported here, TTRwt amyloid structures adopt different morphologies according to the experimental conditions used, in particular pH, in agreement with diverse morphologies found in vivo [64]. TTR amyloid formed at pH 2.0 and pH 7.4 (heat-induced) is characterized by unbranched fibrillar structures, being particularly long at pH 2.0, while amyloid formed at pH 4.4, or pH 2.0 and pH 4.4 subsequently adjusted to pH 7.4, presents a mixture of different species including fibrils, spherical aggregates, and amorphous aggregates. All these types of structures have been previously observed ex vivo, isolated from different organs and biological tissues.

The heat-induced aggregation protocol at pH 7.4 has significant advantages over the other protocols, since it uses a low protein concentration, the pH is maintained at physiological conditions, and the amyloid morphology is characterized by long unbranched fibrillar structures. On the other hand, the pH 4.4 protocol also has advantages, namely it is the least time-consuming, uses low protein concentration, the temperature is physiological, amyloid morphology is heterogeneous which is also found in vivo, and turbidity assays to follow aggregation/disaggregation are well adapted to a 96 micro-well plate format. When the morphology of aggregated species is an important factor to be considered, a judicious choice of the aggregation protocol must be taken into account: (i) for long unbranched amyloid fibrils, the aggregation protocol at pH 2.0 is recommended; (ii) for shorter unbranched amyloid fibrils, the aggregation protocol at pH 7.4; (iii) for a mixture of amyloid fibrils, spherical, and amorphous aggregates, the aggregation protocol at pH 4.4 or pH 4.4 followed by adjustment to pH 7.4; and (iv) for a mixture of spherical and amorphous aggregates, the aggregation protocol at pH 2.0 followed by adjustment to pH 7.4 is recommended.

To characterize the main molecular interactions between TTR fibrils/aggregates and potential small molecule disruptors, we used ^1^H STD NMR. Applying this methodology to the well-known disruptor doxycycline, hydrogen atoms involved in the interaction with preformed TTRwt aggregates/fibrils prepared by different aggregation protocols were identified. These experiments provide qualitative information on the epitope map of the fibrillar disruptor responsible for the interaction with the aggregates/fibrils, which in turn is of paramount importance in the rational design of new fibril disruptor compounds. The results of the ^1^H STD NMR showed that doxycycline interacts differently with TTRwt amyloid structures formed in different experimental conditions and, consequently, with different amyloid morphologies. The disruptive effect of doxycycline on preformed TTRwt amyloid fibrils in vitro was also evaluated for the first time, and we demonstrated that doxycycline disassembles preformed TTRwt aggregates and fibrils in all the aggregation conditions tested. As shown in Figure 2, the activity of doxycycline in disrupting amyloid species of TTRwt was higher in mature amyloid fibrils than in other amyloid morphologies. These findings are in complete agreement with in vivo results showing that doxycycline is more efficient at disrupting already formed fibrillar deposits [42].

In conclusion, we demonstrated here that it is possible to follow disaggregation of preformed TTRwt amyloid using different aggregation protocols. Following up on this work, we will take advantage of these findings to screen a library of small molecules as potential TTRwt amyloid fibril disruptors. In addition, we will also test these compounds in different TTR amyloidogenic variants and other amyloid systems, such as the amyloid-beta peptide and lysozyme, which are implicated in Alzheimer’s disease and lysozyme amyloidosis [1], respectively.

## Figures and Tables

**Table 1:** Experimental conditions of three TTRwt aggregation protocols.

pH	Temperature	Incubation Period	Protein Concentration	Stirring
2	25 °C	At least 1 week	80 μM and then diluted to 3.6 μM	No
4.4	37 °C	3 days	3.6 μM	No
7.4	60 °C	6 days	3.6 μM	No

**Table 2:** Characterization of TTRwt amyloid species formed by different aggregation protocols.

Aggregation Protocol	Morphology of the Aggregates and Fibrils	Length/Diameter of Aggregated Species	Thioflavin-T Assay	Effect of pH Adjustment to pH 7.4	
pH 2.0	Long, unbranched, mature fibrils	50 to 170 nm / 5 to 9 nm	Positive	Alterations in the size and secondary structure of fibrils	Fibrils remain ThT positive
pH 4.4	Mixture of spheroid structures, amorphous aggregates, and short unbranched fibrils	Spheroid aggregates (4 to 6 nm); fibrils (25 nm / 4–5 nm)	Positive	Slight effect on size, secondary structure, and morphology of aggregates to more amorphous and less fibrillar species	Fibrils remain ThT positive
pH 7.4	Unbranched fibrils	10 to 50 nm/4 to 6 nm	Positive	Not necessary	

**Table 3:** Secondary structure estimation by Circular Dichroism using the web server BeStSel.

Aggregation Protocol	pH 2.0	pH 2.0 → pH 7.4	pH 4.4	pH 4.4 → pH 7.4	pH 7.4 (60 °C)	pH 7.4 (37 °C)
α-Helix	0.14	0.03	0.07	0.08	0.11	0.09
β-Sheet	0.43	0.32	0.32	0.24	0.4	0.39
Turn	0.09	0.13	0.16	0.17	0.11	0.13
Unordered	0.34	0.52	0.45	0.51	0.38	0.39

## Supplementary Materials

The following supporting information can be downloaded at: https://www.mdpi.com/article/10.3390/ijms23010391/s1.
