# Supplementary material for: Searching for the Best Transthyretin Aggregation Protocol to Study Amyloid Fibril Disruption
Source: Int J Mol Sci. 2021 Dec 30;23(1):391. doi: 10.3390/ijms23010391 (PMC8745744; doi:10.3390/ijms23010391)
Supplement: Supplementary file 1 [file ijms-23-00391-s001.zip › ijms-1383729-supplementary.pdf]

Supplementary Materials

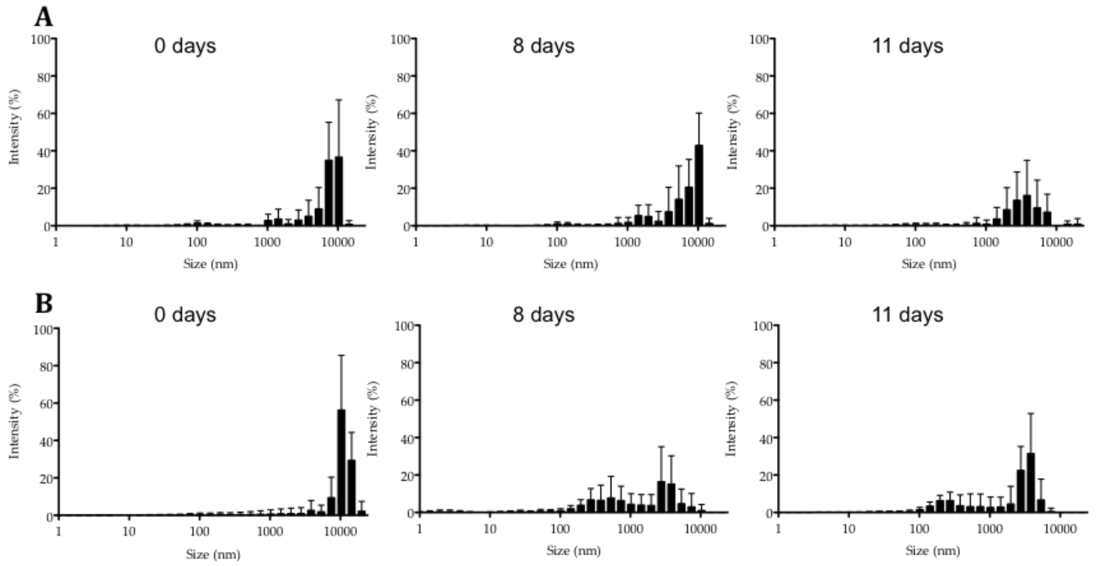

Figure S1: DLS analysis of the effect of pH adjustment to 7.4 in preformed TTRwt fibrils assembled at pH 2.0 with 0.1 M NaCl. (A) SDP (size distribution processor) analysis represented as size distribution by intensity of TTRwt fibrils (3.6 μM) assembled at pH 2.0 with 0.1 M NaCl at 0, 8 and 11 days; (B) SDP analysis represented as size distribution of TTRwt fibrils assembled at pH 2.0 with 0.1 M NaCl followed by pH adjustment to 7.4 at 0, 8 and 11 days.

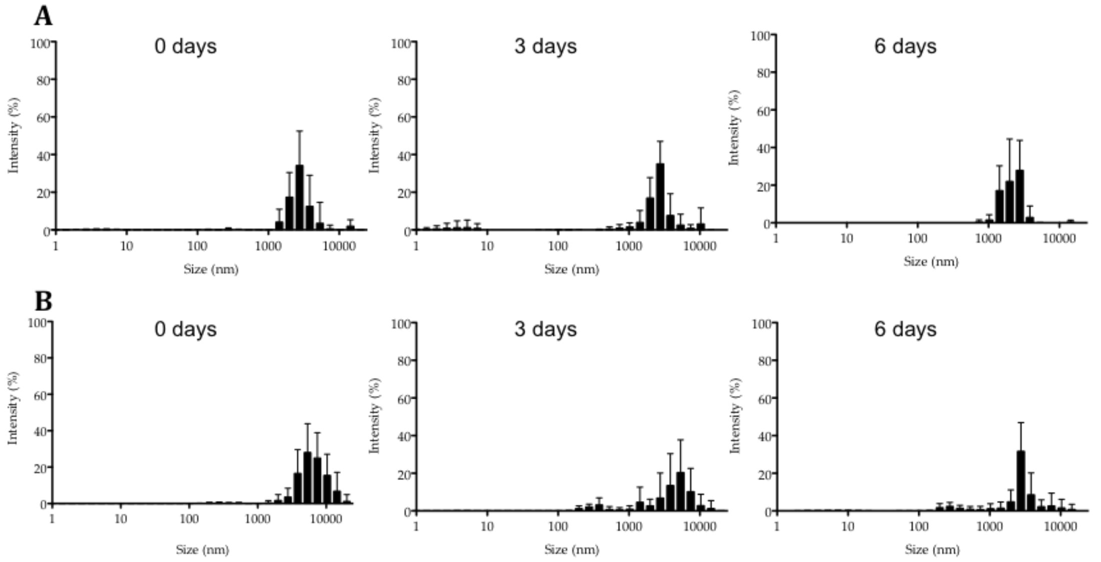

Figure S2: DLS analysis of the effect of pH adjustment to 7.4 in preformed TTRwt fibrils assembled at pH 4.4. (A) SDP analysis represented as size distribution of TTRwt fibrils (3.6 μM) assembled at pH 4.4 at 0, 3 and 6 days; (B) SDP analysis represented as size distribution of TTRwt fibrils assembled at pH 4.4 followed by pH adjustment to 7.4 at 0, 3 and 6 days.

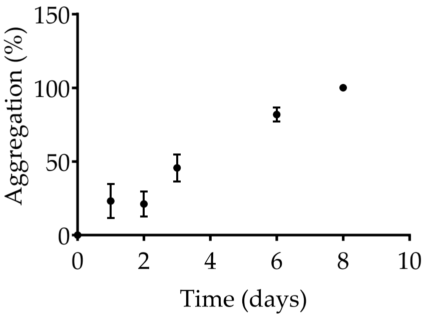

Figure S3: TTRwt fibril assembly induced by heating at pH 7.4 monitored by turbidity. Samples were incubated at 60 °C for the first 6 days and maintained at 37 °C in the last two.

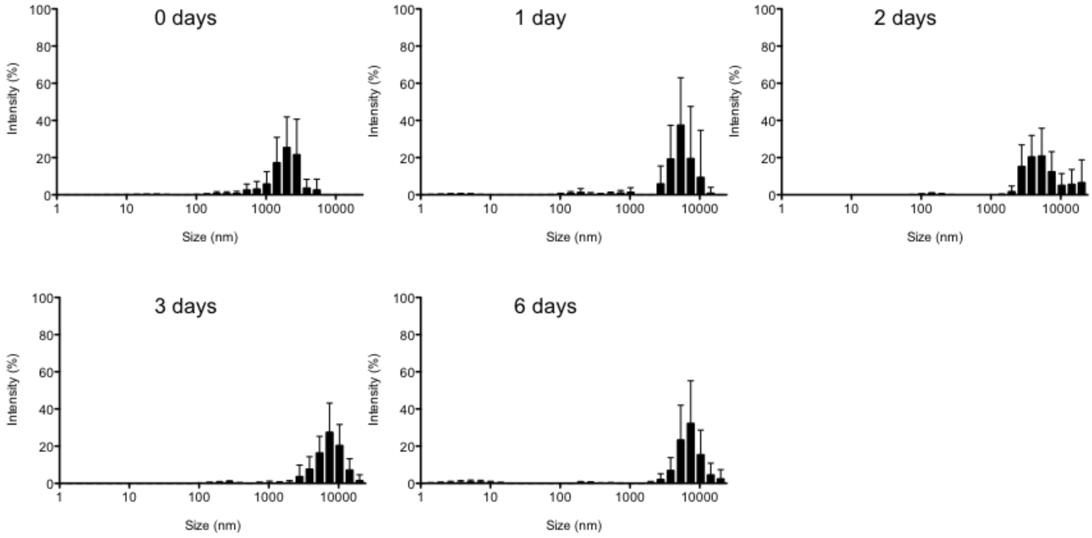

Figure S4: DLS analysis of TTRwt fibril assembly at pH 4.4. Measurements were taken at 0, 1, 2, 3 and 6 days and performed at 37 °C. SDP analysis is represented as size distribution by intensity.

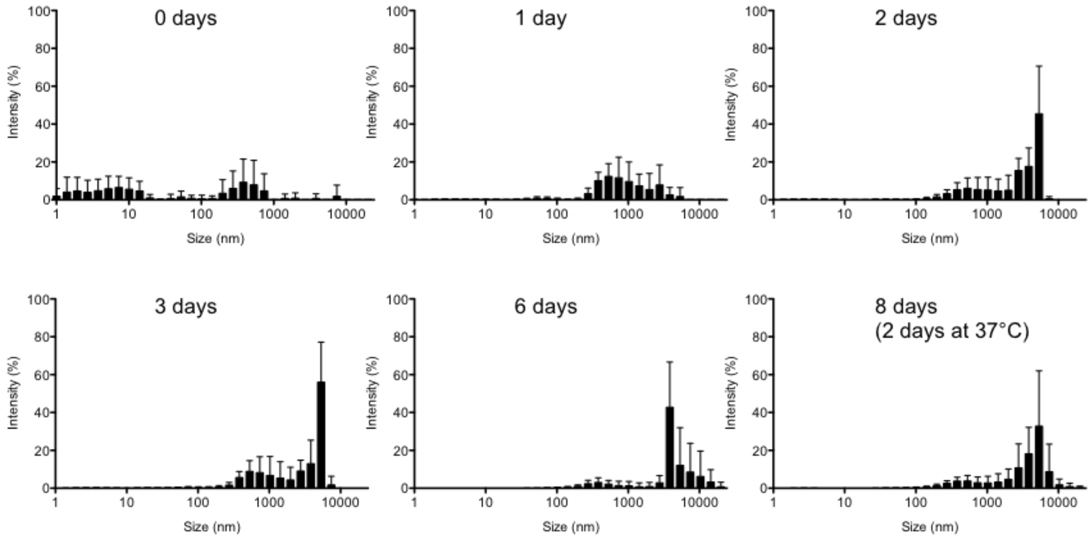

Figure S5: DLS analysis of TTRwt fibril assembly induced by heating at pH 7.4. TTRwt (3.6 μM) was incubated at 60 °C for the first 6 days and maintained at 37 °C in the last 2 days, and measurements were performed at 37 °C. SDP analysis is represented as size distribution by intensity.

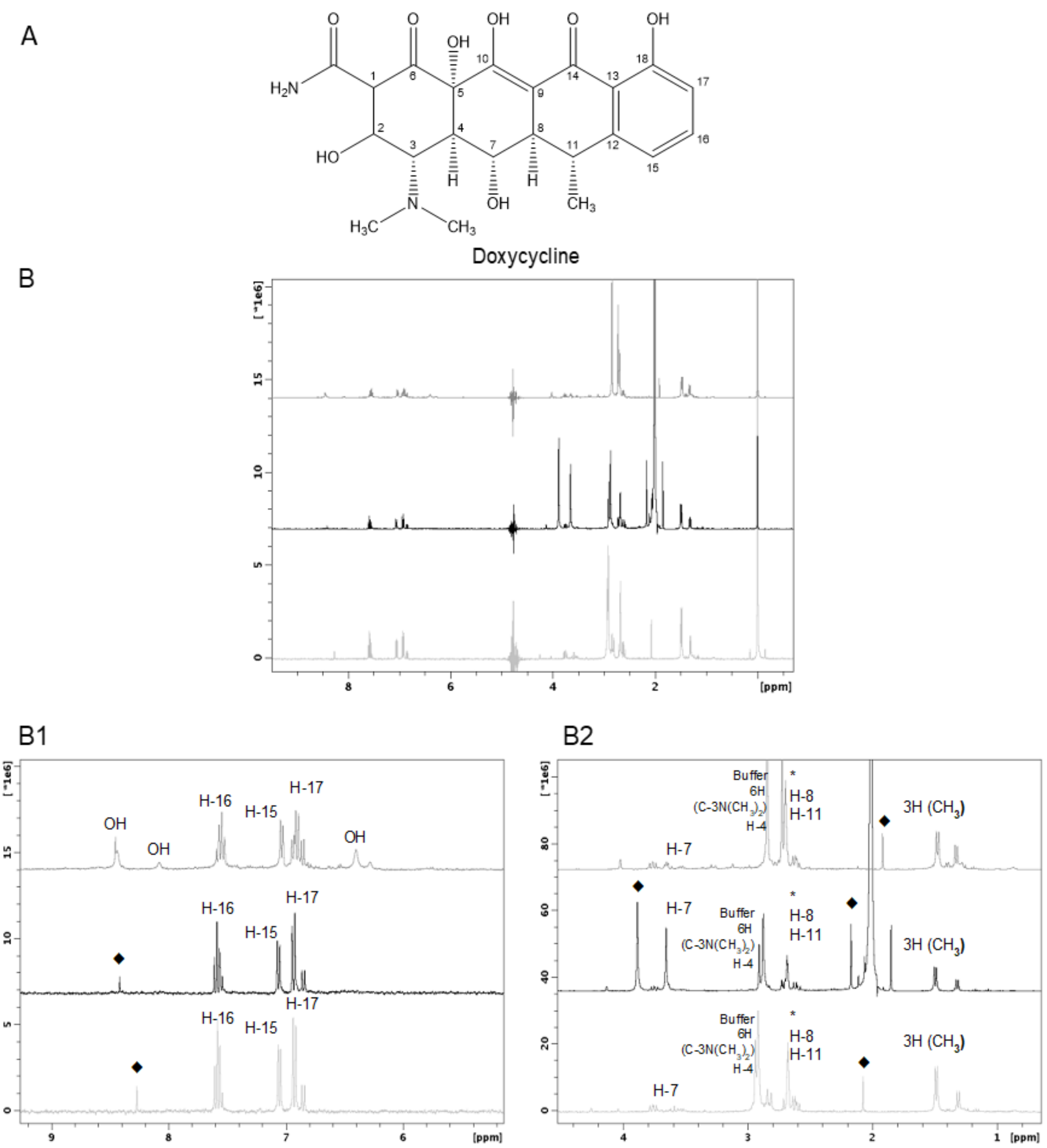

Figure S6: Doxycycline chemical stability at different pH analysed by NMR. Molecular structure of doxycycline (A) and ^{1}H NMR spectrum (B) with spectral expansions (B1 and B2) of doxycycline (1 mM) in phosphate saline buffered (PBS) pH 7.4 (grey), acetate buffer pH 4.4 (black) and 10 mM HCl with 0.1 M NaCl pH 2.0 (light grey). The signal at 0 ppm corresponds to the TSP (3-(trimethylsilyl)propionic acid) reference, the asterisk to DMSO-d_6 and the symbol ◆ to sample impurities.
